# Case Report—Secondary Antibody Deficiency Due to Endogenous Hypercortisolism

**DOI:** 10.3389/fimmu.2020.01435

**Published:** 2020-07-07

**Authors:** Jelena Sarcevic, Claudia Cavelti-Weder, Christoph T. Berger, Marten Trendelenburg

**Affiliations:** ^1^Division of Internal Medicine, University Hospital Basel, University of Basel, Basel, Switzerland; ^2^Division of Endocrinology, Diabetes and Metabolism, University Hospital Basel, Basel, Switzerland; ^3^Translational Immunology, Department of Biomedicine, University of Basel, Basel, Switzerland; ^4^Clinical Immunology Laboratory, Department of Biomedicine, University of Basel, Basel, Switzerland

**Keywords:** hypogammaglobulinemia, lymphocytopenia, immune deficiency, secondary antibody deficiency, endogenous hypercortisolism

## Abstract

Therapeutic corticosteroids have an immunosuppressive function involving several pathways, including lymphocytopenia and hypogammaglobulinemia. While these effects have been well-described in patients that received corticosteroids for therapeutic reasons, the effects of endogenous corticosteroids on the immune system are less well-understood. Here, we describe a 21-year old patient with hypercortisolism due to an ACTH producing thymic tumor. In this patient, we observed a decrease in some of the immunoglobulin classes, and in specific B and T cell populations that resembled effects caused by corticosteroid treatment. IgG levels were restored following treatment and normalization of the hypercortisolism.

## Introduction

Corticosteroids are an essential part of the treatment of allergic and inflammatory diseases. Corticosteroid therapy leads to leukocytosis with lymphopenia, monocytopenia, and eosinopenia, as well as decreasing immunoglobulin levels. In 1973, Butler and Rossen (1) published the first study describing hypogammaglobulinemia in humans that had received methylprednisolone. They also observed a less marked and more transient decrease in total IgA and IgM. Since then, several reports confirmed this observation in asthmatic patients treated with oral corticosteroids (2–6), and additionally observed a temporary increase in IgE in some of the patients (5). Low levels of IgG may persist even after discontinuation of steroid treatment (2). Interestingly, there was no correlation between serum IgG and the duration of treatment, only between daily dose and hypogammaglobulinemia (4, 6, 7). Decreased IgG production as well as an increase in IgG catabolism have been suggested to contribute to corticosteroid-induced hypogammaglobulinemia (8).

More recently, Wirsum et al. (7) investigated hypogammaglobulinemia in patients with giant cell arteritis and polymyalgia rheumatica treated with corticosteroids. They described isolated IgG deficiency without a decrease of other immunoglobulin classes. This hypogammaglobulinemia was associated with a decrease in naïve and transitional B cells while memory B cells, class-switched B cells, and plasmablasts were preserved. In this way, corticosteroid-treated patients differ from patients with primary antibody deficiency due to common variable immunodeficiency (CVID), in which typically a decrease in at least two classes of immunoglobulins and a decrease of class-switched B cells, memory B cells and plasmablasts occurs. While these patterns provide a clear distinction between secondary antibody deficiency due to corticosteroid therapy and CVID, the effects of endogenous hypercortisolism on immunoglobulin levels and lymphocyte (sub)populations remain elusive. Here, we studied the characteristics of immunoglobulin levels and the distribution of T and B cell (sub)populations in a patient with endogenous hypercortisolism due to an ACTH-producing tumor.

## Presentation of Case

A 21-year-old male Caucasian presented to our hospital with the chief complaint of weight gain (around 11 kilograms in 3 weeks) and lower leg edema, which had started 3 weeks ago. He was also suffering from fatigue, lack of attentiveness, intermittent palpitations, and a facial rash. Furthermore, he had a history of panic attacks for which he was treated with methylphenidate (Ritalin®) for 3 months before his admission. There were no previous surgical or medical interventions and he had no family history of endocrine or immunologic disease. He was an occasional smoker but drank no alcohol. Physical examination showed lower leg edema, facial edema, and arterial hypertension ranging between 141/82 and 172/100 mmHg before treatment. The initial blood tests showed leucocytosis with neutrophilia and lymphocytopenia, hypokalaemia, elevated liver enzymes (1.5× of upper norm) and morning glucose levels ranging between 6 and 9 mmol/l. Based on the patient's history and physical examination, an endocrine disorder was suspected. An endocrinological workup revealed low testosterone and LH-levels and elevated ACTH and cortisol levels (no suppression in low dose (1 mg) dexamethasone suppression test). An MRI of the head showed no micro- or macroadenoma of the pituitary gland, so ectopic ACTH production was assumed. CT-scan showed an anterior mediastinal tumor measuring 4 × 3 × 3 cm in size, bilateral hypertrophic adrenal glands as well as lesions in the left humeral head and the left third rib, suggestive of metastases of a primary thymic tumor. A transsternal thymectomy was performed. Histological examination showed a thymic neuroendocrine tumor consisting of 20% of a large cell neuroendocrine carcinoma and 80% of an atypical carcinoid. Further analyses showed an expression of somatostatin receptor 2a (SSTR2A) in 15% of the tumor cells, 30% expression of ACTH, no expression of PDL1 and a beta-catenin mutation.

**Figure 1:**
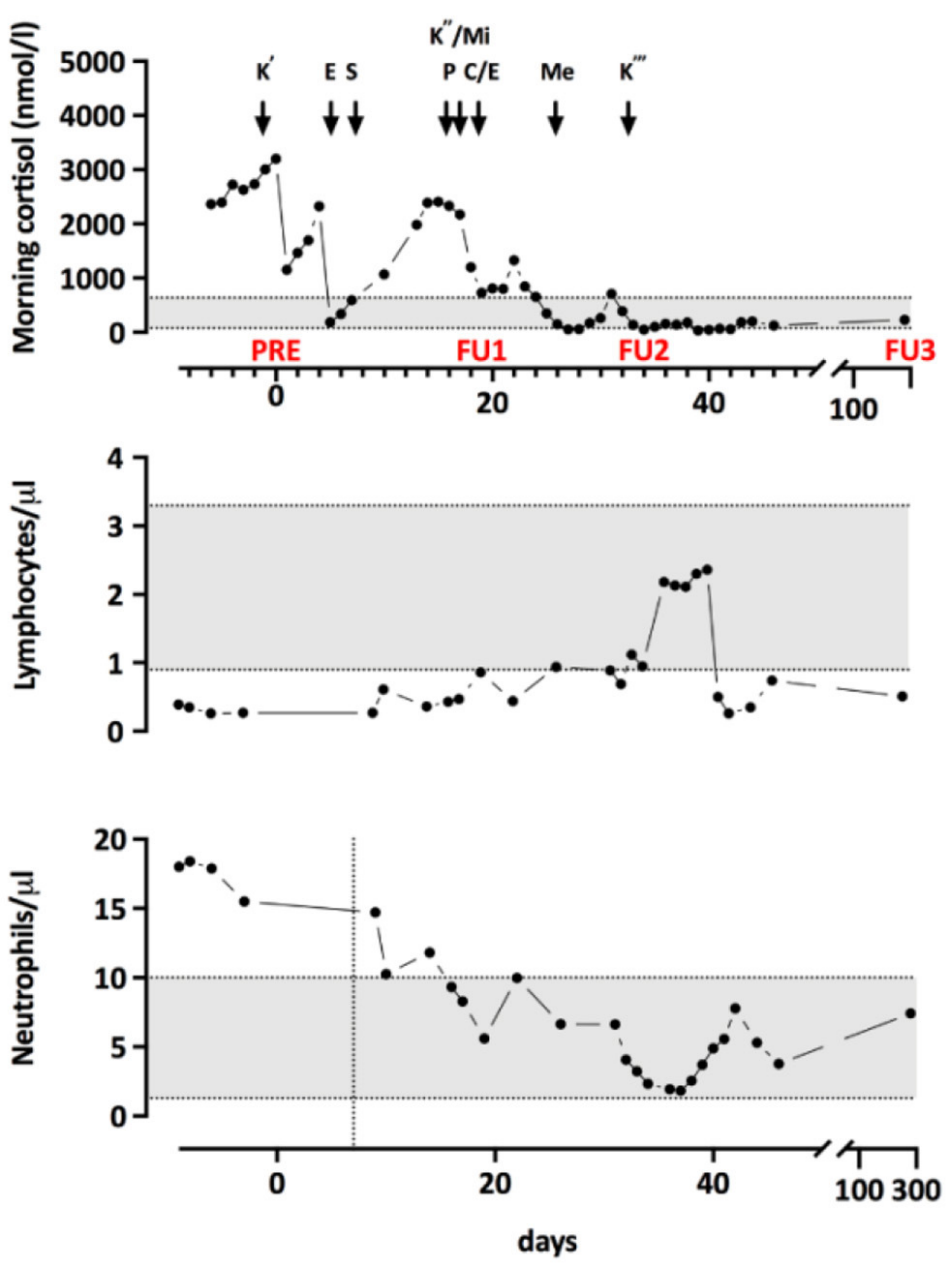
Morning cortisol levels throughout treatment. K', start of Q5 Ketoconazole treatment; E, intravenous Etomidate; T, transsternal thymectomy; K”, Restart of Ketoconazole with both Mifepristone (Mi) and Pasireotide (P). Due to not satisfying response, E was restarted and chemotherapy with Cisplatin and Etoposide (C/E) began. Me, Metopirone treatment; K”', Replacement of E by Ketoconazole. PRE, FU1, and FU2 indicate the timepoints of the immunological measurements.

To lower the level of cortisol, treatment with Ketoconazole was initiated. Because of an unsatisfactory response, additional treatment and a more complex regimen became necessary. The progression of morning cortisol levels under the different therapies is shown in Figure 1. Additionally, he was started on an adjuvant chemotherapy (four 3 week cycles of Cisplatin/Etoposide) and radiotherapy of the mediastinum and the bone metastases, which started during the 4th cycle. Three weeks after the completion of the radiotherapy and 4 months after diagnosis, a PET-CT scan showed a slight progression of the disease with new bone metastases. Two months after completion of the radiotherapy, radiopeptide therapy with ^177^Lu-Dotatote was started and the patient underwent three rounds of treatment with 2 months between every round.

**Figure 2:**
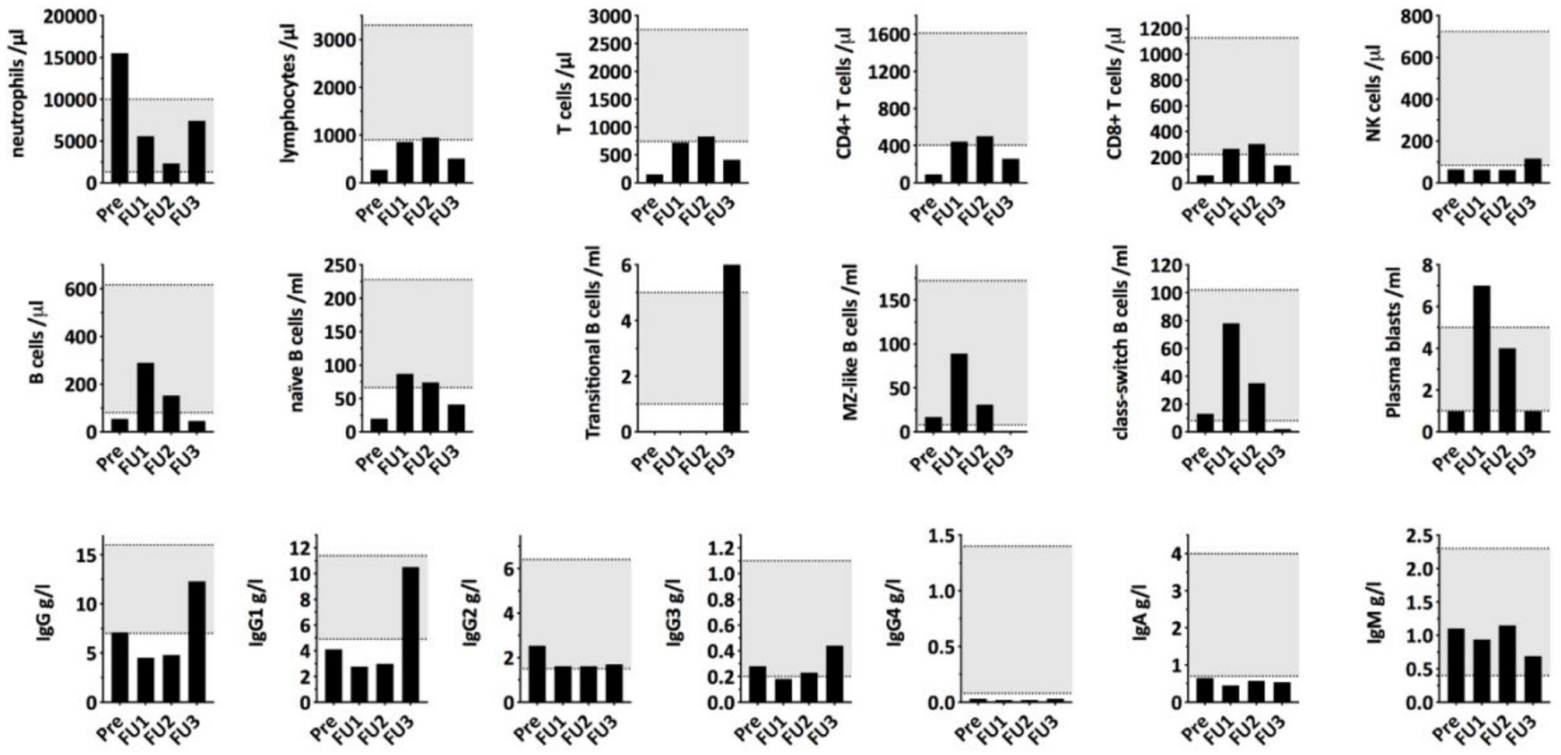
Serum immunoglobulin concentrations and lymphocyte (sub)populations compared to morning cortisol levels during cortisol lowering treatment. PRE, first measurement before starting Ketoconazole treatment; FU1, second measurement 12 days after the surgical removal of the thymic tumor/start of chemotherapy with Cisplatin/Etoposide; FU2, third measurement 15 days later and 27 days after thymectomy with cortisol levels being in the normal range. FU3, fourth measurement 321 days after first measurement and after undergoing chemotherapy, radiotherapy, and during radiopeptide therapy.

To investigate the immunological consequences of hypercortisolism in our patient, more in-depth, repetitive measurements of serum concentrations of immunoglobulin classes and IgG subclasses as well as quantification of lymphocyte populations and B cell subpopulations were performed. Immunological alterations were compared to endogenous morning cortisol levels as determined during the monitoring of cortisol lowering treatment (Figure 2). As shown in Figure 2, IgA, IgG1, and IgG4 were decreased and he had reduced frequencies of CD4+ and CD8+ T cells and NK cells in his peripheral blood. The B cell subpopulations showed a specific reduction in naïve and transitional B cells. Upon treatment of the hypercortisolism, leukocyte counts normalized in parallel to the decreasing cortisol levels. While T cell and B cell counts partially recovered, NK cells and transitional B cells remained decreased. Total IgG (especially IgG1 and IgG4) and IgA remained low. The immune dysregulation showed partial recovery during the subsequent follow-up almost a year after diagnosis and despite the chemotherapy and radiotherapy. Specifically, total IgG and transitional B cells had recovered. Due to chemotherapy induced lymphocytopenia, most remaining immune subsets remained low: the patient continually had low levels of T-cells, NK-cells, B-cells, naïve B-cells, Class-switched B-cells, and plasmablasts. The reduced total IgA and IgG4 persisted, and IgE levels were not detectable.

Next, we assessed the pathogen-specific immunity to vaccines. The patient was vaccinated according to the Swiss childhood vaccination plan and was up to date with all immunizations. The prolonged hypercortisolemia apparently had no negative effect on the already established pathogen-specific immunity. The vaccine-induced IgG levels against measles, tetanus and hepatitis B, as well as the naturally acquired varicella-immunity were preserved despite the hypercortisolism and chemotherapy (Table 1). After completion of the chemotherapy, we assessed the vaccine-response to polysaccharide vaccines [i.e., the Polysaccharide pneumococcal vaccine (PPV) Pneumovax-23] as a functional test of the T-cell independent B cell response. Post vaccination titers in all seven tested pneumococcal serotypes increased to protective levels, indicating a robust B cell function *in vivo*, despite the still present profound lymphocytopenia at the time of vaccination.

**Table 1:** Pathogen-specific immunity and vaccine response to pneumococcal polysaccharide immunization.

**Antigen**	**Reference**	**Levels post-therapy**	
Hepatitis B surface Ag	(>100)	229.00 IU/l	
Varicella	(>150)	2,818 mlU/ml	
Measles	(>13.5)	39.8 AU/ml	
Tetanus Toxoid	(>100)	327 IU/l	
**Pneumococcal serotype**		**Pre-PPV23**	**Post-PPV23**
Serotype 4	(>0.3)	0.4 mg/l	2.1 mg/l
Serotype 6B	(>0.3)	<0.3 mg/l	>5.0 mg/l
Serotype 9V	(>0.3)	<0.3 mg/l	0.8 mg/l
Serotype 14	(>0.3)	0.5 mg/l	>5.0 mg/l
Serotype 18C	(>0.3)	<0.3 mg/l	1.2 mg/l
Serotype 19F	(>0.3)	1.1 mg/l	4.2 mg/l
Serotype 23F	(>0.3)	<0.3 mg/l	0.7 mg/l

Since the total immunoglobulin levels showed a remarkable recovery during the follow-up and despite chemotherapy, we currently suggest assessing the lymphocyte populations and total-immunoglobulin levels twice a year or in the case of any new infections.

## Discussion

Until now, the effect of hypercortisolism on immunoglobulin levels and lymphocyte populations has primarily been studied in patients receiving corticosteroids (most commonly prednisone) for therapeutic reasons. To our knowledge, this case is the first detailed analysis of immunoglobulin levels and lymphocyte populations in a patient with endogenous hypercortisolism. Our patient showed a decrease in IgA, IgG1, IgG4, and IgM as well as low NK cells and low transitional and naïve B-cells. This constellation resembles the findings of Wirsum et al. in patients with long-standing corticosteroid therapy (7). However, in contrast to the mostly isolated IgG deficiency that was transient in 25% of the patients, our patient showed a persistent decrease in IgG1, IgG4, and IgA despite normalizing morning cortisol levels. Hamilos et al. (4) observed a decrease in all IgG subclasses, IgA and IgM in some asthmatic patients treated with oral corticosteroids. The differences between our patient and data from studies in glucocorticoid treated patients might be due to different effects of therapeutic prednisone vs. endogenous hypercortisolism on the immune system, but more studies are required to exclude patient-specific causes.

Of note, our patient did not develop any severe and opportunistic infections even during chemotherapy, radiotherapy or radiopeptide therapy with ^177^Lu-Dotatote. Previous case reports suggested an increased risk of severe infection in patients with endogenous hypercortisolism, but the clinical significance of hypogammaglobulinemia in these patients regarding the risk of infection remains controversial. Graham and Tucker (9) described a series of 23 patients with opportunistic infections in endogenous Cushing's syndrome. They found a correlation between the degree of hypercortisolism and risk of infection and considered extreme hypercortisolism as an “immunologic emergency,” but detailed data concerning the association of observed alterations of the immune system and the risk and severity of infection are scarce. Hamilos et al. (4) found no significant association between the number of infectious episodes and hypogammaglobulinemia in asthmatic patients treated with corticosteroids. Last, reduced immunoglobulin levels and low lymphocyte numbers may not necessarily reflect a compromised function of the immune system. Thus, not only quantitative but also functional analyses of the immune system in patients with endogenous hypercortisolism are necessary.

In summary, we show the effects of endogenous hypercortisolism on immunoglobulin levels and lymphocyte populations, which resemble effects observed and described in patients receiving corticosteroids for therapeutic reasons. Preexisting pathogen-specific immunity was preserved, and upon suppression of the hypercortisolism IgG serum levels were restored and B cells were functional *in vivo*. The phenotype associated with hypercortisolism, as well as the clinical relevance of these immunocompromising effects need to be determined in cohort studies to exclude patient-specific causes.

## Ethics Statement

Written informed consent was obtained from the patient for the publication of data included in this article according to the requirements of the responsible ethics committee (i.e., Ethikkommission Nordwestschweiz) for medical case reports.

## Author Contributions

JS was involved in the patient care and responsible for data aggregation and primary writing of the case report. CC-W was involved in the patient care and provided essential expert knowledge for the interpretation of the data. CB was involved in analyzing, interpreting, and presenting the immunological data. MT supervised the case report and was involved in data analysis and interpretation. All authors contributed to the article and approved the submitted version.

## Conflict of Interest

MT is recipient of a project grant of the Swiss National Science Foundation (grant No. 310030_172956/1) and has research collaborations with F. Hoffmann-La Roche, The Novartis Institutes for BioMedical Research (NIBR), and Idorsia Pharmaceuticals (all Basel, Switzerland). The remaining authors declare that the research was conducted in the absence of any commercial or financial relationships that could be construed as a potential conflict of interest.
